# Individualized automated planning for dose bath reduction in robotic radiosurgery for benign tumors

**DOI:** 10.1371/journal.pone.0210279

**Published:** 2019-02-06

**Authors:** Linda Rossi, Alejandra Méndez Romero, Maaike Milder, Erik de Klerck, Sebastiaan Breedveld, Ben Heijmen

**Affiliations:** Department of Radiation Oncology, Erasmus MC Cancer Institute, Rotterdam, The Netherlands; North Shore Long Island Jewish Health System, UNITED STATES

## Abstract

**Object:**

To explore the use of automated planning in robotic radiosurgery of benign vestibular schwannoma (VS) tumors for dose reduction outside the planning target volume (PTV) to potentially reduce risk of secondary tumor induction.

**Methods:**

A system for automated planning (AUTOplans) for VS patients was set up. The goal of AUTO- planning was to reduce the dose bath, including the occurrence of high dose spikes leaking from the PTV into normal tissues, without worsening PTV coverage, OAR doses, or treatment time. For 20 VS patients treated with 1x12 Gy, the AUTOplan was compared with the plan generated with conventional, manual trial-and-error planning (MANplan).

**Results:**

With equal PTV coverage, AUTOplans showed clinically negligible differences with MANplans in OAR sparing (largest mean difference for all OARs: ΔD2% = 0.2 Gy). AUTOplan dose distributions were more compact: mean/maximum reductions of 23.6/53.8% and 9.6/28.5% in patient volumes receiving more than 1 or 6 Gy, respectively (p<0.001). AUTOplans also showed smaller dose spikes with mean/maximum reductions of 22.8/37.2% and 14.2/40.4% in D2% for shells at 1 and 7 cm distance from the PTV, respectively (p<0.001).

**Conclusion:**

Automated planning for benign VS tumors highly outperformed manual planning with respect to the dose bath outside the PTV, without deteriorating PTV coverage or OAR sparing, or significantly increasing treatment time.

## Introduction

Stereotactic radiosurgery is an increasingly used option for management of patients with benign vestibular schwannoma (VS) tumors [1–5]. However, an increased risk of secondary tumor formation associated with radiation exposure is well established [6–10]. Therefore, in radiotherapy, dose outside the planning target volume (PTV) should be avoided as much as possible, especially for patients with a long life expectancy.

High delivery accuracy can be obtained with the robotic system due to real-time image-guided tracking, allowing small PTV margins [11, 12], and non-coplanar treatment [13].

Currently, treatment plans in radiotherapy are generally generated in an interactive trial-and-error process in which the planner tries to steer the treatment planning system (TPS) towards generation of an acceptable solution (“manual planning”). This may be a time consuming process and the resulting plan quality may heavily depend on the skills and experience of the planner and on the available planning time and software. The potential of automated treatment planning for both enhancement of plan quality and drastic reduction in planning time, as alternative to manual planning, has been shown in many studies [14–23].

However, the existing literature on automated planning is focused on reduction of high doses in OARs or enhancement of PTV dose. To the best of our knowledge, this is the first study focusing on the use of automated planning for overall reduction of dose bath. The study was inspired by observing the inability, in manual planning, to optimally reduce the dose bath, and the planning time investment in getting a possible improvement, as systematically tried.

In our institution, a system for fully automated, multi-criterial treatment plan generation has been developed, including optimization of beam directions. For this study, this system was used as a pre-optimizer for the clinical TPS, that comes with the robotic treatment unit to automatically generate deliverable plans for VS patients (AUTOplan). We investigated whether automated planning could reduce the dose bath compared to manually generated plans (MANplan), while not deteriorating dose delivery to the PTV or OARs, and keeping treatment times comparable.

## Materials and methods

### Patients and manual planning (MANplan)

Contoured CT scans of 20 vestibular schwannoma patients, previously treated at our department with the CyberKnife (CK) robotic treatment unit (Accuray Inc, Sunnyvale, USA), were included in the study. All patient-related information was fully anonymized prior conducting the research. According to the regulations of the Ethics Committee of Erasmus MC no ethical approval for this retrospective study was needed as there was no impact on treatment and the applied patient data.

The gross tumor volume (GTV) was defined as the volume of contrast enhancement on the T1-weighted MRI, after CT/MRI fusion. No margins were applied for planning (GTV = PTV). The average tumor size was 2.9 cc (1.0-7.3 cc). Delineated OARs were brainstem, trigeminal nerves, area of virtual facial nerve, optic nerves, chiasm, cochlea (when clinically relevant), pituitary gland, and eyes.

For planning, tumor coverage (>98% of the volume receiving 100% of the prescribed dose) had highest priority, while strictly fulfilling OAR Dmax constraints and high dose conformality around the target. Furthermore, the goal was to maximally reduce the dose bath, including minimizing dose spikes. Various planning strategies were clinically used to control this dose spillage, based on planners’ preferences and experience. All MANplans were generated in Multiplan [24] clinical TPS (Accuray Inc, Sunnyvale, USA) that comes with the CK, with the Iris variable aperture collimator [25], avoiding diameters smaller than 10 mm and larger than 40 mm. CK full head node path was used for a total 179 non-coplanar available beam directions.

Patients were treated with a single fraction of 12 Gy, prescribed at the 80% isodose.

### Automated planning (AUTOplan)

Clinically deliverable CK plans for VS patients were generated in a two-step process:

1. For each patient, a pre-plan was automatically generated with the in-house developed Erasmus-iCycle TPS [20, 21, 23, 26]. Plan generation with Erasmus-iCycle is driven by a so-called wish-list, describing all planning constraints and objectives. The latter have ascribed priorities for steering the multi-criterial plan generation, resulting in clinically favorable trade-offs between all treatment objectives. Generated plans are Pareto-optimal. For this study, a wish-list was made for VS plan generation, minimizing dose spillage from the PTV while not deteriorating PTV and OAR dose, or substantially increasing the treatment time. Erasmus-iCycle and wish-list building have been extensively described in the literature [13, 20].
2. The Erasmus-iCycle plan generated in step *i* was used to automatically create a patient-specific planning template for subsequent automatic plan generation by the clinical TPS Multiplan. The planning template contained the planning constraints to ensure generation in Multiplan of a clinically deliverable plan that mimicked the original Erasmus-iCycle plan.

This two-step approach was developed as for practical and regulatory reasons Erasmus-iCycle cannot be used directly for clinical plan delivery.

A similar two-step approach is currently clinically used at Erasmus MC for automated VMAT planning for prostate cancer, head and neck cancer, cervical cancer, and advanced lung cancer, using the Monaco clinical TPS (Elekta AB, Stockholm, Sweden) in the second step [21]. A distinct difference between VS autoplanning for robotic treatment and automated VMAT planning is the inclusion of non-coplanar beam-angle selection for VS.

All generated AUTOplans were based on the same planning protocol, the same choice from Iris collimators, node set, and the same dose prescription as used for clinical MANplan generation (above).

To minimize dose spillage in AUTOplans, the template for automated planning with Multiplan (step *ii* above) contained individualized Dmax constraints for shells at distances of 1, 3, and 5 cm from the PTV. For each patient, these shell constraints were obtained from the Erasmus-iCycle plan, generated in step *i*. In Erasmus-iCycle, the maximum doses at these shells were minimized, while respecting all hard planning constraints and the (higher) priorities for adequate PTV coverage and OAR sparing. AUTOplan generation in Multiplan in step *ii* was performed in high resolution with intensive treatment time reduction.

### Automated planning with fixed shell constraints

As described above, individually optimized shell constraints, derived from the Erasmus-iCycle plan, were used in the second step of AUTOplan generation with the Multiplan TPS. In an additional analysis, we investigated whether it was possible to use for all patients the same Dmax constraints instead of patient-specific values. This study was performed to understand how patient-specific constraints impact plan quality, keeping all other parameters the same, or, opposite, to see if it was possible to use equal values for all patients without losing in plan quality.

For each shell, the population-fixed constraint was calculated as the average of the patient-specific Dmax values found in the Erasmus-iCycle plans of the 20 study patients. Then, for each of the patients, a second AUTOplan was generated, using the fixed constraints. In the remainder of the paper, the second AUTOplans with fixed constraints are referred to as *fAUTOplans*.

### Plan comparisons

Prior to comparisons of AUTOplans with MANplans and fAUTOplans with AUTOplans all plans were rescaled to a tumor V12Gy of 98%, when achieved (in line with the clinical planning protocol). Plans were compared using OAR near-maximum doses, D2%, conformity index (CI), and treatment time. To evaluate dose bath and spikes, patient volumes V_*D*_ with *D* up to 10 Gy were assessed, as well as D2%, of shells from 1 to 7 cm away from the tumor, for spillage. Two-sided Wilcoxon signed-rank tests were used to analyze plan differences, using p<0.05 for statistical significance.

## Results

### AUTOplan vs. MANplan

All MAN- and AUTOplans were clinically acceptable with tumor coverage >98% (98.3±0.1%) while fulfilling all OAR constraints (Table 1). Treatment times were comparable; 36.1±5.0 min and 38.2±4.1 (p = 0.008) for MAN- and AUTOplans, respectively, allowing plan comparison without unacceptable treatment time difference bias.

**Table 1:** For all 20 patients, mean values for automatically generated plans (AUTOplans) and manually generated (MANplans). Bold values represent the statistically significant differences as p<0.05 with the Wilcoxon’s signed-rank test.*CI = Conformity Index.

		AUTOplans	MANplans	
		Mean ± SD	Mean ± SD	
PTV	V12Gy	98.3 ± 0.1	98.3 ± 0.1	%
	CI*	1.2 ± 0.1	1.2 ± 0.1	
Brainstem	D2%	9.7 ± 1.5	9.8 ± 1.4	Gy
	Dmean	**2.2** ± 0.6	**2.4** ± 0.8	Gy
Trigeminal Nerve	D2%	11.7 ± 1.0	11.8 ± 0.8	Gy
Facial Nerve	D2%	14.0 ± 0.2	14.0 ± 0.3	Gy
L Optic Nerve	D2%	0.1 ± 0.0	0.2 ± 0.1	Gy
R Optic Nerve	D2%	0.1 ± 0.0	0.2 ± 0.1	Gy
Chiasm	D2%	0.3 ± 0.1	0.3 ± 0.1	Gy
Cochlea	D2%	11.6 ± 1.2	11.8 ± 1.1	Gy
Pituitary	D2%	0.3 ± 0.1	0.4 ± 0.1	Gy
L Eye	D2%	0.1 ± 0.0	0.1 ± 0.0	Gy
R Eye	D2%	0.1 ± 0.0	0.1 ± 0.0	Gy
Patient	V1Gy	**271.2** ± 142.9	**347.6** ± 162.5	cc
	V2Gy	**68.0** ± 32.2	**84.7** ± 44.8	cc
	V3Gy	**36.2** ± 16.6	**44.0** ± 22.9	cc
	V4Gy	**24.1** ± 10.9	**28.8** ± 14.9	cc
	V6Gy	**14.3** ± 6.5	**16.2** ± 8.3	cc
	V8Gy	**9.6** ± 4.6	**10.3** ± 5.3	cc
	V10Gy	6.2 ± 3.3	6.4 ± 3.4	cc
PTV Shell 1cm	D2%	**3.7** ± 0.5	**4.9** ± 0.8	Gy
PTV Shell 2cm	D2%	**2.0** ± 0.4	**2.6** ± 0.5	Gy
PTV Shell 3cm	D2%	**1.4** ± 0.3	**1.7** ± 0.3	Gy
PTV Shell 5cm	D2%	**1.0** ± 0.2	**1.2** ± 0.2	Gy
PTV Shell 7cm	D2%	**0.8** ± 0.2	**1.0** ± 0.2	Gy
Treatment Time		**38.2** ± 4.0	**36.1** ± 4.8	min
MU		**4899** ± 704	**5716** ± 996	
Nodes		**56.1** ± 10.8	**66.6** ± 21.1	
Beams		**161.8** ± 24.2	**121.8** ± 29.6	

CIs for AUTOplans (1.16±0.06) were slightly better than for MANplans (1.18±0.09), but the difference was not significant (p = 0.4).

As for PTV coverage, MAN- and AUTOplans were comparable in terms of OARs sparing. For all OARs, the mean difference in near-maximum dose was below 0.2 Gy and statistically not significant. Only the brainstem Dmean was on average slightly lower for the AUTOplans, which was statistically significant, 2.2±0.7 Gy vs. 2.4±0.8 Gy, p = 0.02.

**Fig 1:**
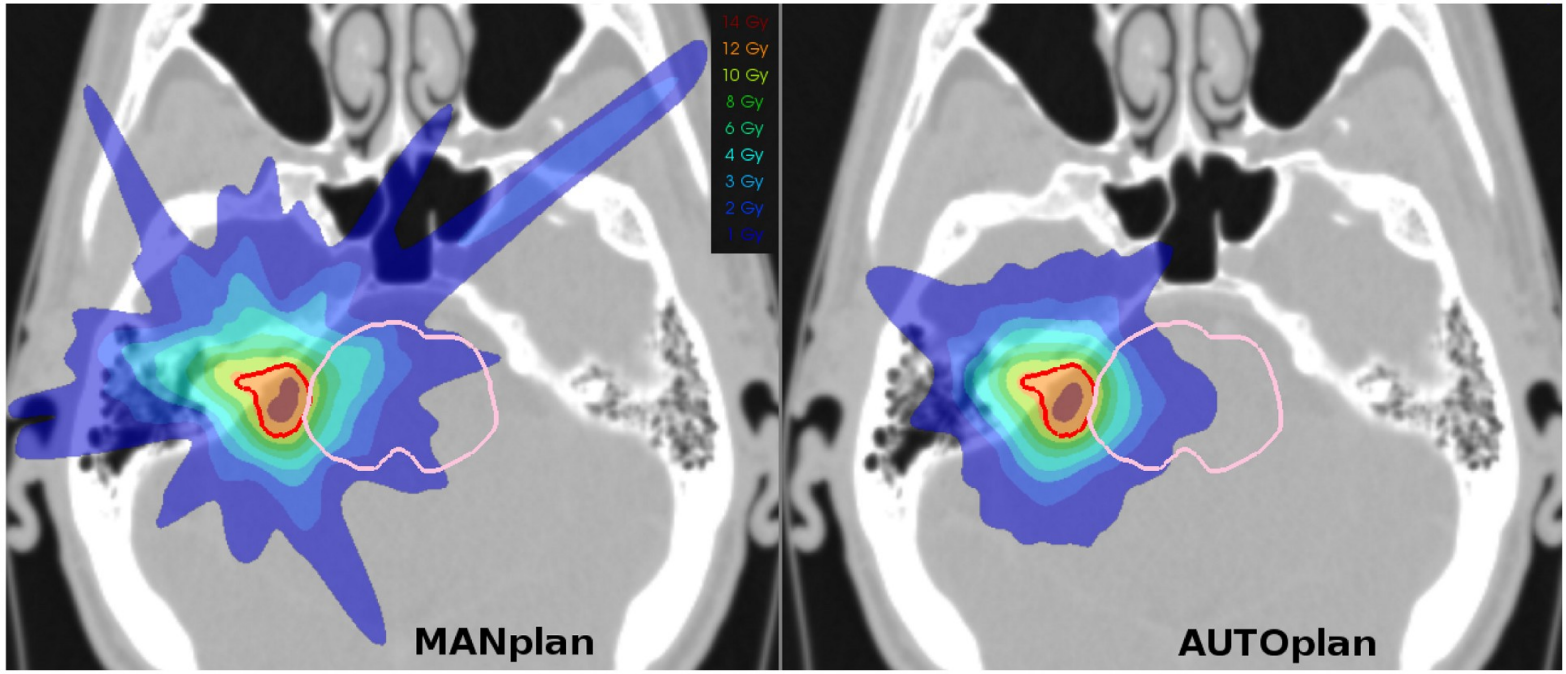
Axial dose distributions for an example patient. The AUTOplan (right) shows a reduced dose bath, with also smaller dose spikes, than the MANplan (left). Reduction in brainstem dose is also visible. (Red contour = PTV, pink contour = Brainstem).

**Fig 2:**
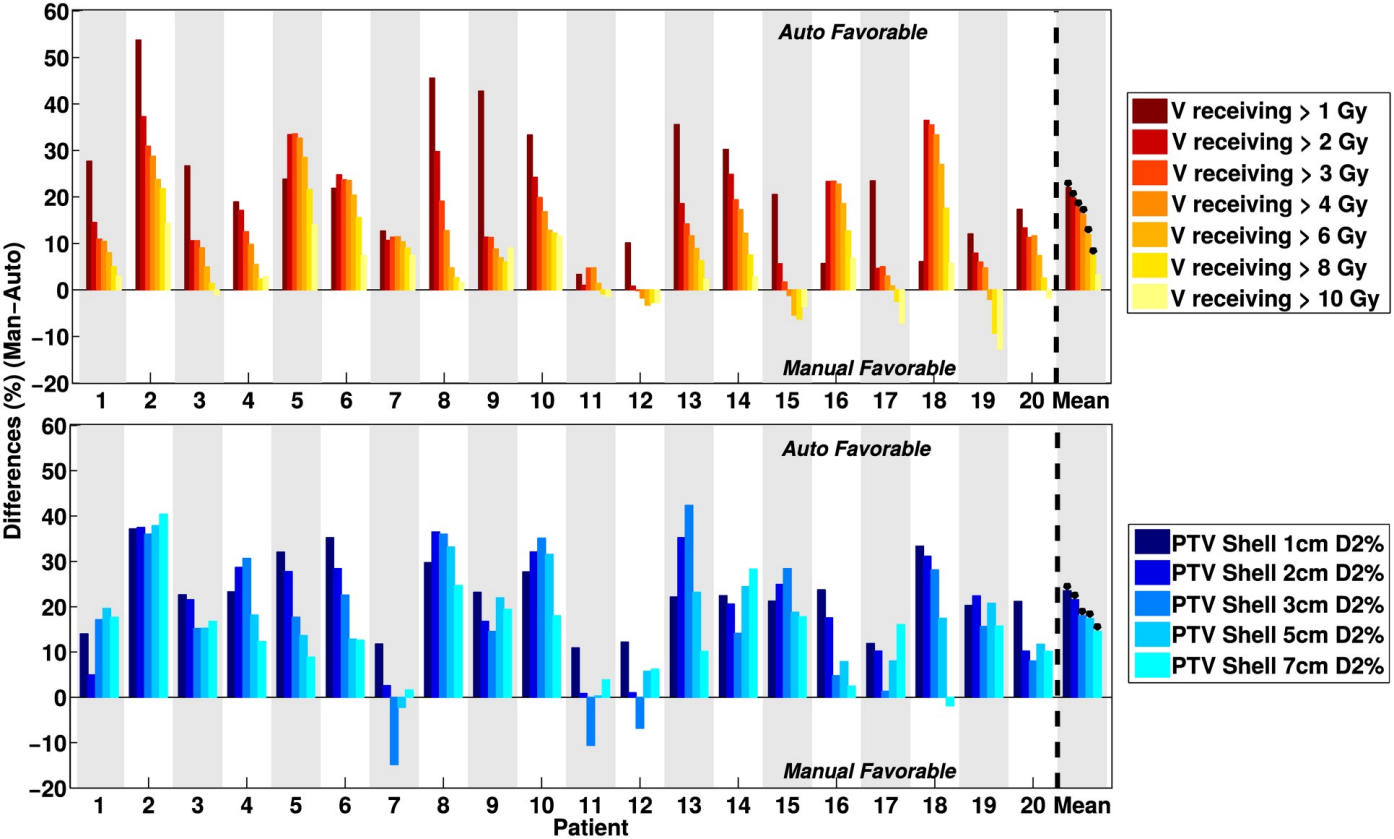
Differences between AUTOplans and MANplans in dose spillage. Positive differences reflect higher quality for the AUTOplan. Upper panel: differences in patient volumes receiving more than 1, 2, 3, …, 10 Gy. Lower panel: differences in D2% for shells at 1, 2, …, 7 cm away from the tumor. For the individual patients, differences were calculated as (MANplan − AUTOplan)/MANplan * 100. The last column shows population mean differences and their statistical significance marked with * (p<0.05).

Dose bath was substantially reduced in the AUTOplans (see Fig 1 for example). Both patient volumes receiving dose and dose spikes were reduced with autoplanning compared to manual planning. For most patients, volumes V_*D*_ receiving more than *D* Gy were smaller in the AUTOplans, as visible in the upper panel of Fig 2 and Table 1. Population-average percentage V_*D*_ reductions for D = 1, 2, 3, 6, 8, and 10 Gy were 23.6%, 17.5%, 15.3%, 13.5%, 9.6%, 6.1% and 2.9%, respectively (all p<0.005, apart from V10Gy with p = 0.06). AUTOplans also had reduced near-maximum doses in the shells at 1, 2, 3, 5 and 7 cm from the PTV, mostly reflecting smaller spikes and closer to the tumor. Average reductions of shell D2% with AUTOplans were 22.8%, 20.5%, 16.8%, 16.7% and 14.2%, respectively (all p<0.001), as visible in the lower panel of Fig 2 and Table 1.

AUTOplans used less MU (4899 vs. 5716) and nodes (56.1 vs. 66.6), but more beams (161.7 vs. 121.8), all p<0.004.

### fAUTOplan vs. AUTOplan

Table 2 shows for each patient the Dmax for the shells at 1, 3, and 5 cm from the PTVs, as obtained from plan optimization with Erasmus-iCycle, and used as individualized shell constraints in Multiplan optimization in the second phase of the AUTOplan generation. The presented population mean values were used for all the patients to generate the fAUTOplans.

**Table 2:** For each patient, Dmax values for shells at 1, 3 and 5 cm from the PTV as derived from the Erasmus-iCycle plan, and population mean values with standard deviations. Patient-specific Dmax values were used as constraints in AUTOplan generations, while the mean values were used for fAUTOplan generation.

Patient-specific constraints	Shell 1 cm Dmax [cGy]	Shell 3 cm Dmax [cGy]	Shell 5 cm Dmax [cGy]
Pt 1	427	156	112
Pt 2	374	140	106
Pt 3	430	157	117
Pt 4	423	173	138
Pt 5	458	171	133
Pt 6	426	155	120
Pt 7	428	168	129
Pt 8	370	146	98
Pt 9	390	123	88
Pt 10	344	108	74
Pt 11	423	170	105
Pt 12	417	174	137
Pt 13	368	135	107
Pt 14	475	181	124
Pt 15	357	119	84
Pt 16	536	210	162
Pt 17	396	165	123
Pt 18	471	198	167
Pt 19	522	181	134
Pt 20	486	200	149
**Mean±SD**	**426±53**	**162±27**	**120±25**

Using population average shell constraints instead of the individualized constraints (Table 2) for plan generation in Multiplan (fAUTOplan) resulted in an unacceptable PTV coverage for 5 out of 20 patients (Table 2, patients 14, 16, 18, 19 and 20), with a minimum of 92.3% instead of 98%, due to too tight shell constraints.

For the remainder of the patients, with equal PTV coverage, there were no statistically significant differences between fAUTOplans and AUTOplans in CI, mean and near-maximum OAR doses, patient volumes V_*D*_ receiving more than D Gy (D≤10 Gy), and D2% at shells from 1-7 cm from the PTV.

## Discussion

Several studies have observed superiority of automated plan generation compared to conventional, interactive trial-and-error planning regarding enhancement of PTV dose, or reduction of OAR doses [14, 16, 21, 26, 27]. Apart from plan quality advantages, automated planning did always result in drastic reductions in planning workload.

Especially for patients with a long life expectancy, such as VS patients, dose outside the PTV should be maximally avoided to reduce the risk for secondary tumor induction. To the best of our knowledge, this is the first paper on automated planning that primarily aims at the overall reduction of low and high doses outside the PTV of a benign tumor. To this purpose, a system was set up and configured for fully automated non-coplanar plan generation for robotic stereotactic treatment of VS patients.

The project was started after observing frequent problems with controlling or improving dose bath and spikes from the PTV in manual planning (see Fig 1a). In this study we have demonstrated that automated planning could reduce the dose bath, without worsening PTV coverage or OAR sparing, or substantially increasing treatment time. Moreover, due to the automation, there was no workload in the plan generation.

As explained in the M&M section, final AUTOplans were generated with the commercial TPS that comes with the robotic treatment unit. Therefore, this TPS is indeed able to generate highly compact plans, with minimal dose delivery outside the PTV and avoiding dose spikes. In the autoplanning procedure, the individualized shell constraints, used in the commercial TPS to maximally avoid dose spillage, were automatically generated using a pre-optimization with our in-house developed multi-criteria optimizer (step *i*, M&M section). In conventional manual planning, shell constraints are defined for each patient in a trial-and-error procedure. Then, finding the optimal constraint values for individual patients is often impractical, because it is too time consuming to be feasible in clinical routine.

The presented workflow for AUTOplan generation of highly compact dose distributions is likely to be applicable also for other benign tumors, which will be a topic for further research.

To the best of our knowledge, this is the first study to systematically investigate a possible reduction in dose bath while mantaining treatment technique. Integral dose reduction is a reported outcome from many plan comparison studies [28–31], beside the OARs evaluation, as well as different metrics have been proposed [32, 33], reflecting the clinical relevance. However a systematic dose bath reduction can not be easily investigated without a consistent planning work flow such as the one used in this study, including the dose bath improvement without loosing quality in the PTV and OAR dose distributions.

For 5/20 patients, the applied population-based, fixed shell constraints used for generating the fAUTOplan were too tight, resulting in an unacceptably low PTV coverage. No correlation with PTV size or other features was found. For the other 15 patients, the fAUTOplan was similar in quality as the AUTOplan. This does however not necessarily imply that in clinical routine, acceptable high quality plans would have been generated for the latter patients, using the fixed constraints. fAUTOplans were generated with an automated planning workflow and it is uncertain whether using the population-based constraints with manual planning would have resulted in high quality plans for these 15 patients. This could have been dependent on the complexity of the individual cases and on the skills of the involved planners. Anyway, compared with automated planning, the workload would have increased.

On the longer term and to serve the radiotherapy community, there is a clear need for more advanced commercial treatment planning systems that are faster and/or facilitate automated plan generation.

## Conclusion

Compared to conventional planning, automated plan generation for robotic treatment of patients with a benign vestibular schwannoma tumor could to a large extent reduce low to high dose outside the PTV while maintaining acceptable tumor coverage and similar OAR dose, and keeping delivery time comparable.

## Supporting information

S1 File The data underlying the findings in present study. (XLS)
